# Microbially assisted recording of the Earth's magnetic field in sediment

**DOI:** 10.1038/ncomms10673

**Published:** 2016-02-11

**Authors:** Xiangyu Zhao, Ramon Egli, Stuart A. Gilder, Sebastian Müller

**Affiliations:** 1 Department of Earth and Environmental Sciences, Ludwig-Maximilians University, Munich 80333, Germany; 2 Central Institute of Meteorology and Geodynamics (ZAMG), Division Data, Methods and Models, Vienna 1190, Austria

## Abstract

Sediments continuously record variations of the Earth's magnetic field and thus provide an important archive for studying the geodynamo. The recording process occurs as magnetic grains partially align with the geomagnetic field during and after sediment deposition, generating a depositional remanent magnetization (DRM) or post-DRM (PDRM). (P)DRM acquisition mechanisms have been investigated for over 50 years, yet many aspects remain unclear. A key issue concerns the controversial role of bioturbation, that is, the mechanical disturbance of sediment by benthic organisms, during PDRM acquisition. A recent theory on bioturbation-driven PDRM appears to solve many inconsistencies between laboratory experiments and palaeomagnetic records, yet it lacks experimental proof. Here we fill this gap by documenting the important role of bioturbation-induced rotational diffusion for (P)DRM acquisition, including the control exerted on the recorded inclination and intensity, as determined by the equilibrium between aligning and perturbing torques acting on magnetic particles.

## Abstract (web summary)

Sediments record variations of the Earth's magnetic field via the alignment of magnetic grains during and after deposition, yet the role of post-depositional processes remains unclear. Here, the authors present experiments showing how microbially-induced bioturbation controls the alignment process.

## Graphical abstract

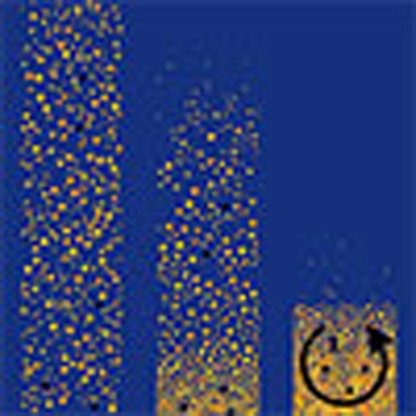

The acquisition of a depositional remanent magnetization (DRM) or post-depositional remanent magnetization (PDRM) in sediment can be understood by following the path of settling particles in the water column and within the biologically mixed uppermost layer, until their orientation becomes fixed during consolidation^1^,^2^,^3^ (Fig. 1a). A net magnetization^4^ is generated by particles with a magnetic moment *m* when they rotate toward the local Earth's field *B* under the action of the magnetic torque *τ*_m_=*mB*. In the water column and at the sediment–water interface, complete alignment is obstructed by particle aggregation^3^,^5^, hydrodynamic forces^6^ and rolling^7^, so that DRM intensity depends on the strength of *B*. A PDRM can be acquired after deposition in the upper part of the sediment column. Two main acquisition mechanisms have been proposed. In the first case, PDRM is acquired without disturbance from irreversible rotation of particles for which *τ*_m_ exceeds the torques generated by inter-particle forces^8^. Since these torques are generally much larger than *τ*_m_, only few magnetic particles will be affected, and most of the original DRM remains intact^9^. The second acquisition mechanism relies on particle realignment by random torques associated with bioturbation. Bioturbation has been simulated in the laboratory by stirring water-saturated sediment in the presence of an ambient field, where a relatively strong magnetization is acquired proportional to the applied field^10^,^11^. A major problem with these experiments is that the sediment samples were dried before the magnetization was measured, so one cannot exclude that the magnetic remanence originated from the drying process^12^.

Regardless of the acquisition mechanism, most PDRM models assume that lock-in of magnetization only begins once substantial surface mixing has ceased, that is, below the mixed layer^3^,^13^, so that DRM and PDRM are mutually exclusive or almost so^14^. A different viewpoint arises from a statistical model of PDRM acquisition in the surface-mixed layer^15^. This model considers bioturbation as a rotational diffusion process similar to that of Brownian motion, which occurs in the presence of random inter-particle forces. In this case, particle orientations are governed by the Debye–Smoluchowski equation^16^, whose solution under stationary conditions yields the equilibrium magnetization:


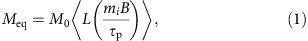


where 〈…〉 denotes the ensemble average over particles with magnetic moments *m*_*i*_, subjected to randomizing torques of mean amplitude *τ*_p_, *M*_0_ is the magnetization corresponding to full alignment, and *L* is the Langevin function. DRM acquisition can be also described by equation (1) with appropriate floc magnetic moments and values of *τ*_p_ representing disturbances at the sediment–water interface. In monodispersed sediments exposed to weak fields (that is, 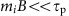), *M*_eq_ is approached exponentially, that is, *M*−*M*_eq_=Δ*Me*^−2*Dt*^, where *M* is the remanent magnetization, Δ*M* the initial value of *M*−*M*_eq_, and *D* the rotational diffusion coefficient associated with bioturbation, which describes the random rotation of individual particles (Supplementary Fig. 1, see Supplementary Note 1 for details). Because smaller particles reorient faster than large ones by virtue of the Stokes–Einstein–Debye relation between rotational and translational diffusion^15^,^17^, the randomization process is controlled by a distribution *p*_r_(*D*) of rotational diffusion coefficients, rather than a single coefficient, in which case:


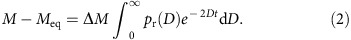


The degree of DRM replacement by a bioturbation-driven PDRM depends on the diffusivity parameter *γ*=*DL*/*ω*, where *L* is the thickness of the mixed layer and *ω* the sedimentation rate^15^. This parameter defines three mixing regimes: (i) a slow (*γ*<0.2) regime where DRM is preserved, (ii) a fast (*γ*>10) regime where DRM is completely replaced by PDRM and (iii) intermediate regimes with partial DRM preservation. Within this framework, DRM and PDRM are products of similar processes under different conditions: DRM coincides with *M*_eq_ during the initial stages of sediment deposition in weak perturbing forces, while PDRM represents the evolution of *M*_eq_ over a much longer time in an environment where stronger forces are required by benthic organisms to move inside the sediment.

We provide here experimental proof for the abovementioned theory based on sediment redeposition experiments. Sediment redeposition is a common technique used to investigate (P)DRM acquisition in the laboratory by recreating the main features of the sedimentation process on much shorter timescales^2^,^3^. Unlike previous redeposition experiments, we use fresh sediment containing abundant living microorganisms, including magnetotactic bacteria^18^,^19^,^20^. We also avoid treatments commonly used to disaggregate sediments and to modify the chemistry of aqueous solutions, which reduce or eliminate the original microorganism communities. Our results confirm the fundamental role of bioturbation for PDRM acquisition inside the sedimentary mixed layer, as well as the progressive replacement of the initially acquired DRM with a PDRM.

## Results

### Sediment redeposition experiments

Organic-rich clay/silt sediment used for this study comes from a small pond near our palaeomagnetic laboratory in Niederlippach, Germany (48°35'15” N, 12°04'43” E). The sediment was collected from the uppermost ∼10 cm below the water–sediment interface and was immediately transferred to glass aquaria at ambient temperature, where a new stably stratified oxygen gradient was re-established within 1 week (ref. 19). Magnetotactic bacteria populations live in the topmost 10 cm, where up to 300 motile cells per μl have been counted in fresh sediment^18^. Cell counts declined by a factor of ∼10 after one year of storage in aquaria^20^. A similar decline is also seen for total bacteria concentrations estimated with the spread plate method. Magnetotactic bacteria are responsible for the accumulation of so-called magnetofossils^21^, that is, chains of single-domain magnetite (Fe_3_O_4_) particles^22^,^23^, which, along with moderate (∼39%) additions of lithogenic magnetic minerals, determine the magnetic properties of the studied sediment. Because of continuous magnetofossil accumulation over time, the magnetic contribution of live magnetotactic bacteria to the sediment magnetization is negligible (Supplementary Note 1), so that temporal variations of bacterial concentrations, for example, during experiments, will not affect sediment properties beyond changes in bioturbation intensity.

Identical sediment samples with different total bacteria concentrations, estimated with the spread plate method, were obtained after laboratory storage in glass aquaria for 1 week (group A: 280 cells per μl), 3 months (group B: 247 cells per μl) and 1 year (group C: 213 cells per μl). Aliquots of group C sediment were subjected to treatments aimed at further reducing microorganism concentrations, that is, sealed storage for 3 months, which removes the natural oxygen gradient^20^ (group D: 207 cells per μl), and the addition of broad-spectrum antibiotics (group E: 126 cells per μl). Macrofauna (for example, polychaete worms) and most meiofauna organisms were removed by wet sieving with a 63-μm mesh before all experiments, so that the effects of bioturbation can be considered homogeneous over the length scale of redeposition experiments. Cell counts obtained with the spread plate method are used here only as a qualitative proxy for the total concentration of benthic microorganisms, since most such microorganisms cannot be cultivated. Furthermore, bioturbation depends also on the type of benthic organisms^24^,^25^, and cannot be quantified by a single parameter. Nevertheless, bioturbation rates can be reasonably assumed to be correlated positively with spread plate results.

**Figure 1:**
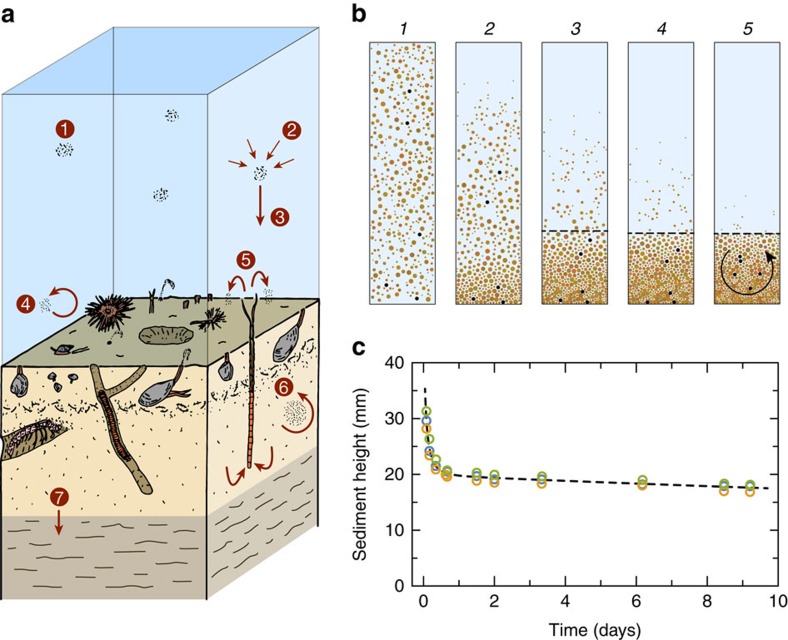
Magnetization acquisition of sediment in nature and in the laboratory. (**a**) Schematic representation of natural processes affecting sedimentary magnetizations (modified from ref. 15). 1: Marine snow, 2: flocculation, 3: settling, 4: sediment resuspension, 5: non-local mixing, for example, by polychaete worms, 6: local (diffusive) sediment mixing leading to particle reorientation and 7: burial in the consolidating layer. (**b**) Schematic representation of sediment redeposition in five different time frames. A homogeneous sediment suspension settles in a magnetic field forming a clear sediment–water interface (dashed line) after some time. The same five particles are highlighted by black dots in each frame. A DRM is acquired by the alignment of magnetized particles in the ambient field during deposition (frames 1–4). This magnetization is stabilized by inter-particle forces developing at contact points (frames 3–4). Sediment mixing (arrow in frame 5) is responsible for particle realignment after deposition and generates a PDRM. Only local (diffusive) sediment mixing is produced in our experiments. (**c**) Height of the sediment–water interface (dots), for three redeposition experiments. The dashed line is a guide for the eye. A nearly stable interface is obtained within the first day.

**Figure 2:**
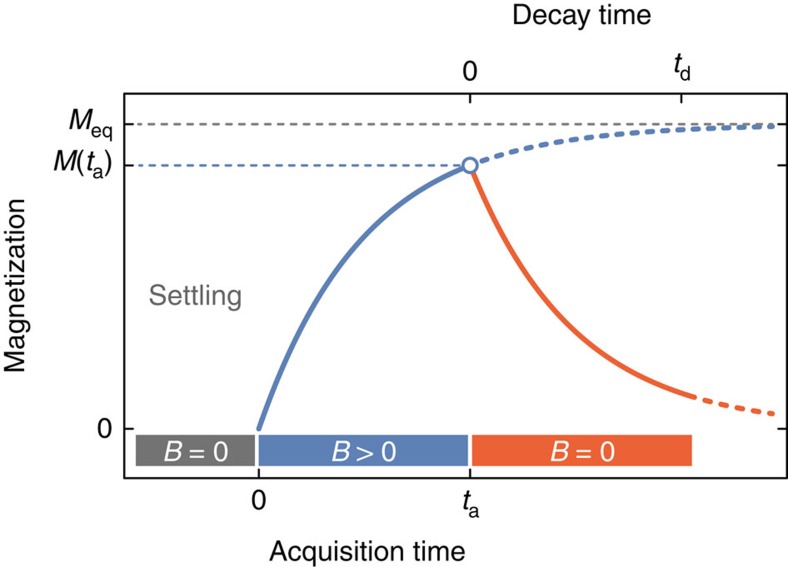
Timing of PDRM acquisition experiments. Sediment is initially settled in a zero field (*B*=0) for ∼5 days. PDRM acquisitions occurs after a controlled field (*B*>0) is applied for *t*_a_≅7 days. During this time, the equilibrium magnetization, *M*_eq_, is approached. Finally, the acquired magnetization, *M*(*t*_a_), is allowed to decay in a zero field for a time *t*_d_. The remanent magnetization during acquisition and decay phases is measured at regular time intervals.

Redeposition experiments were performed in glass vials using sediment material from groups A–E after wet sieving with a 63-μm mesh. Remanent magnetizations were measured with a vertical bore superconducting rock magnetometer^26^. Each vial was prepared by diluting 5 ml of sediment slurry in 10 ml of tap water, sealed, and then vigorously shaken in order to create a homogeneous suspension as a starting condition for all experiments (Fig. 1b). For DRM acquisition experiments, the vials were placed in controlled fields with various intensities and inclinations generated by Helmholtz coils. A clear sediment–water interface formed within 22 h (Fig. 1c). Magnetizations were measured periodically during the experiments by carefully transferring the vials to the magnetometer to avoid mechanical disturbances. A measurement series lasted for ∼10 min, when the vials lay in residual fields of <500 nT inside a magnetically shielded room, before being returned to the controlled field environment. Each DRM (and PDRM) experiment was performed in triplicate (that is, with three independent vials). For the PDRM acquisition experiments, sediment suspensions were allowed to settle in a null field for 5 days, which is sufficient time to obtain a stable sediment column. A controlled field was subsequently applied for ∼7 days (PDRM acquisition), followed again by zero-field conditions for the remaining time to monitor PDRM decay (Fig. 2).

### PDRM acquisition and decay

**Figure 3:**
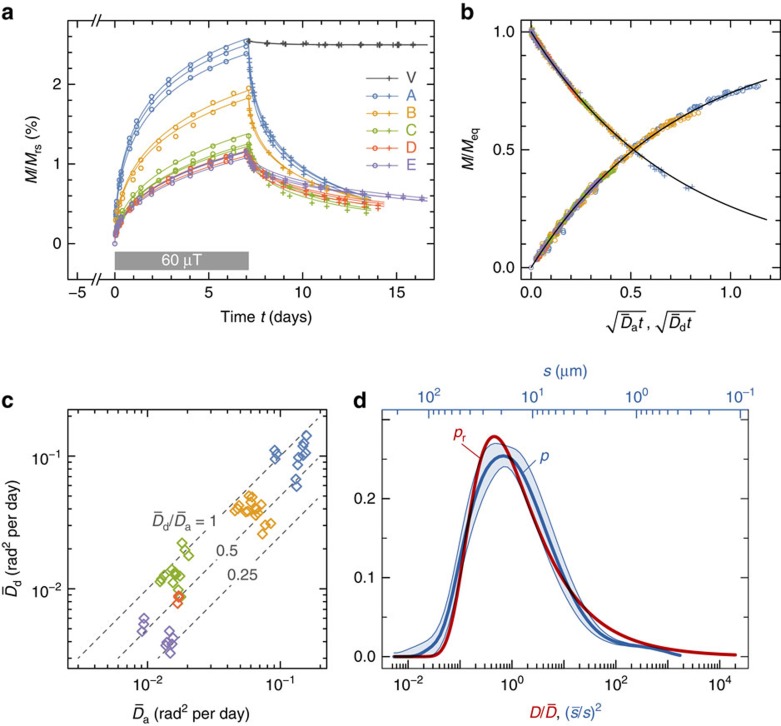
PDRM acquisition results. (**a**) Magnetization versus time for the sediment groups A–E (circles, crosses), after normalization by the mean saturation remanence *M*_rs_. The sediments were deposited in a null field (day −5 to day 0). A 60-μT field with 50° inclination was applied during the next 7 days and was turned off thereafter. Lines are best-fit model curves obtained from equations (2 and 3). Group V designates the PDRM decay due to magnetic viscosity in three fully dried samples, where particle reorientation was not possible. (**b**) PDRM acquisition for the groups A–E in 20-, 40-, 60-, 80-, 100- and 150-μT fields and subsequent zero-field decay. Magnetizations are normalized by the equilibrium PDRM (*M*_eq_) that would be reached after an infinite time. Acquisition/decay times are normalized by the median rotational diffusion coefficients, 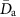 and 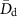, as deduced from least-squares fits of the measured curves (Supplementary Note 1). All data collapse onto a single acquisition/decay curve (black lines), as expected when the rotational diffusion coefficients are identically distributed. (**c**) Median rotational diffusion coefficients 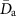 and 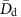 deduced from individual acquisition/decay curves of the sediment groups A–E. 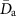 has been corrected for the effect of field intensity (Supplementary Note 1). Dashed lines indicate constant values of 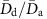, with 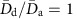 expected for stationary conditions. (**d**) Probability density function *p*_r_ of the rotational diffusion coefficient *D*, reconstructed from the normalized acquisition/decay curves shown in **b**, and the mean distribution *p* of particle diameters *s* for the sediment groups A–E (the shaded band corresponds to ±1 s.d. of 40 measurements). *D* and *s* on the lower axis are normalized by their median values.

**Figure 4:**
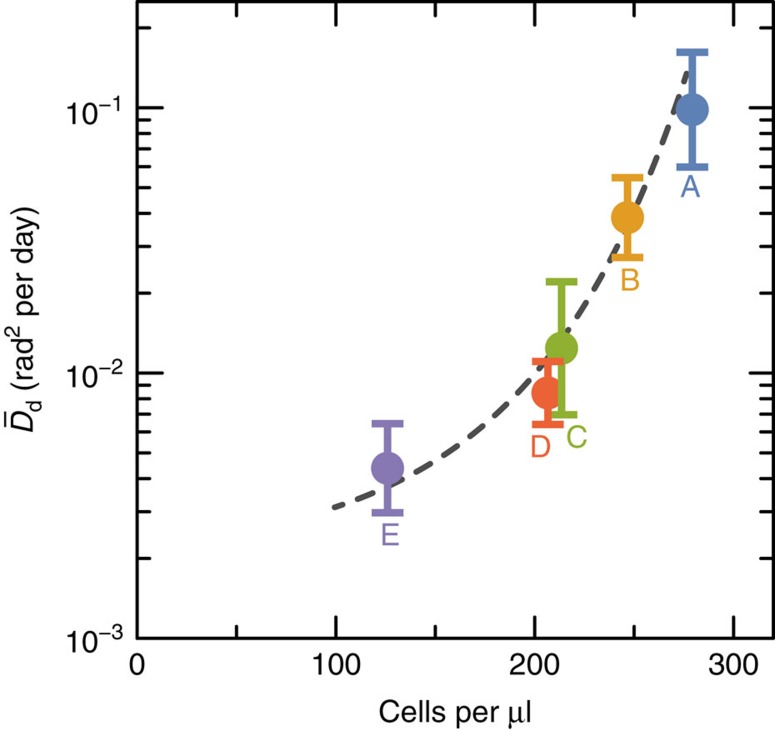
Dependence of the rotational diffusion coefficient on bacterial concentration. Median rotational diffusion coefficient 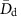 deduced from PDRM zero-field decay curves of the sediment groups A–E (Fig. 3) versus total bacterial concentrations estimated with the spread plate method. The dashed line is a guide for the eye.

PDRM acquisition (Fig. 3a), is due exclusively to mechanical alignment of magnetic particles, as demonstrated by the lack of acquired PDRM and zero-field decay in control samples where particle rotation was hindered by full drying. Acquisition and decay curves were modelled using equation (2) and an empirical distribution


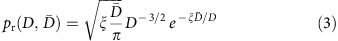


of rotational diffusion coefficients with median 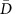 and *ξ*=0.227 (Supplementary Fig. 2). This distribution yields decay and acquisition curves of the type 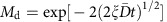 and *M*_a_=1−*M*_d_, respectively (Supplementary Note 1), which reproduce all measurements within experimental errors (Fig. 3b). Differences between median rotational diffusion coefficients 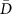 deduced from acquisition and corresponding decay curves are limited mostly to a factor of two (Fig. 3c), which is small compared with the four order of magnitude span of *p*_r_ (Fig. 3d). This means that measured acquisition/decay curves reflect equilibration with the ambient field under nearly stationary conditions. The shape of *p*_r_(*D*) is matched by the size distribution *p*(*s*) of sediment particles (Fig. 3d) if the Stokes–Einstein–Debye relation *D*∝*s*^−2^ of Brownian motion^27^ is assumed to hold inside the sediment (Supplementary Note 1). This means that particle size is an important factor controlling PDRM acquisition rate. Typical values of 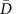 for the groups A–E, on the other hand, correlate positively with measured bacteria concentrations (Fig. 4). This result can only be expected if a diffusion process governs PDRM acquisition at a rate (expressed by 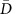) that is, in turn, controlled by bioturbation. Sediment ageing effects can be excluded because the groups C–E are approximately of the same age, yet following the same trend defined by the groups A–C.

### DRM acquisition and decay

**Figure 5:**
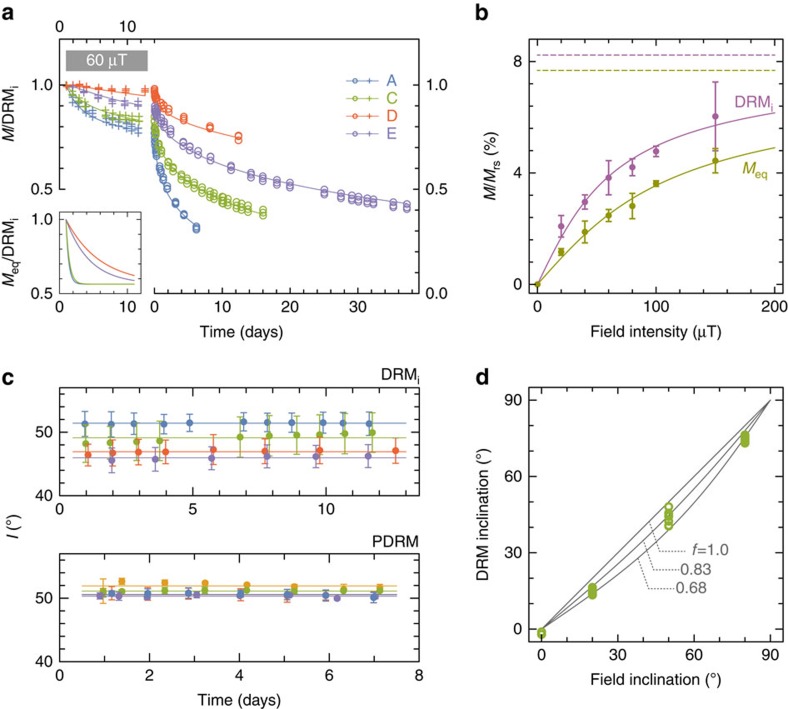
DRM acquisition experiments. (**a**) DRM acquisition in a 60-μT field with 50° inclination for the sediment groups A, C, D and E, as well as subsequent decay in a null field. Zero-field decay curves have been modelled using equations (2 and 3) (solid lines). The median rotational diffusion coefficients deduced from these models were used to fit the DRM acquisition curves, taking into consideration that the initial DRM (that is, DRM_i_) is progressively replaced by a PDRM acquired in the applied field. Because of initial sediment compaction and stabilization, it was assumed that the equilibrium PDRM intensities (*M*_eq_) decrease exponentially from an initial state, characterized by DRM=PDRM at the beginning of deposition (that is, *M*_eq_/DRM_i_=1, see inset), to a final value of *M*_eq_/DRM_i_ matching the measurements shown in **b**. The exponential decrease of *M*_eq_ needed to model the observed DRM changes in time (inset) is faster for sediments containing more bacteria (that is, groups A and C). (**b**) Dependence of DRM_i_ and *M*_eq_ on field intensity. *M*_eq_ was calculated from the PDRM acquisition curves as shown in Fig. 3. Solid lines are least-squares fits with *M*=*M*_0_*S*(*B*/*B*_*0*_), where 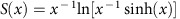 is a suitable approximation of equation (1), *M*_0_ is the magnetization corresponding to full alignment of the magnetic moments (dashed lines), and *B*_0_=15.5 μT and 26.7 μT for DRM_i_ and *M*_eq_, respectively. (**c**) Inclination of DRM_i_ and PDRM. Lines are averages of all experiments from the groups A–E. DRM_i_ inclinations are slightly shallower, especially in sediments containing less bacteria, while no systematic shallowing is observed for the PDRMs. (**d**) Inclination of DRM_i_ in 60-mT fields with 0°, 20°, 50° and 80° inclinations (circles). Solid lines are plots of the inclination shallowing law tan *I*=*f* tan *I*_B_, where *I* and *I*_B_ are the inclinations of DRM_i_ and the applied field, respectively, and *f* is an empirical flattening factor^29^. These results confirm that DRM_i_ has typical properties of a DRM, as seen in the traditional redeposition experiments.

DRM acquisition and its progressive replacement by PDRM was investigated through similar experiments where a magnetic field was applied from the beginning of deposition and maintained for ∼11 days, before measuring its decay in zero field over a period of time (Fig. 5a). Contrary to the redeposition experiments performed with sediment containing no living microorganisms^5^,^28^, the DRM in our experiments decays slowly with time even during continuous field exposure, rather than increasing asymptotically. DRM decay mimics that of PDRM once the field is removed, again following the decay profile described by equations (2 and 3). As in the case of PDRM experiments, magnetic viscosity effects can be ruled out, so that magnetization changes are caused only by particle reorientation. Other aspects of these experiments, such as the recording of shallower than expected inclinations (called inclination shallowing, Fig. 5c,d), mimic those in ‘classic' redeposition experiments^29^ with no living organisms.

The initial DRM intensity and equilibrium PDRM intensity *M*_eq_ depend nonlinearly on the intensity of the applied field for fields exceeding ∼30 μT, which are typical surface field intensities on Earth. This dependence is well-matched by an analytical approximation of equation (1) based on a uniform distribution of *mB*/*τ*_p_, which has been used with success in the past^28^,^29^ (Fig. 5b). Independent fits of the DRM and PDRM data predict similar saturation values (that is, the magnetization caused by full magnetic moment alignment), which means that no grains that carry a DRM are excluded from PDRM acquisition. Therefore, differences between the two magnetization types arise entirely from different degrees of partial magnetic moment alignment. Such differences depend ultimately on *τ*_p_, since the same magnetic moments from the same grains are involved in both cases (that is, the same values of *mB* during DRM and PDRM acquisition). In particular, *τ*_p_ deduced from the PDRM acquisition curve in Fig. 5b is twice as large as for the DRM curve, which confirms that the strength of grain alignment perturbations increase below the sediment–water interface, due to stronger inter-particle forces being overcome by bioturbation. With these data in mind, DRM decay in the presence of a magnetic field can be explained by the fact that the DRM acquired during initial deposition is replaced progressively by a new equilibrium—the PDRM—as *τ*_p_ increases due to the build-up of inter-particle bonds. Because PDRM in weak fields is ∼50% lower than DRM, the net effect is a decrease of the total magnetization. The observed time dependence of DRM in the applied field is reproduced exactly by the PDRM replacement model if *M*_eq_ is assumed to decrease exponentially from an initial state characterized by the equivalency of DRM and PDRM at the beginning of deposition, to a final value matching the PDRM acquisition experiments shown in Fig. 3.

## Discussion

Our experiments confirm that bioturbation is responsible for PDRM acquisition inside the sedimentary mixed layer, eventually replacing the initial DRM if rotational diffusion is fast enough with respect to the mean residence time of particles in this layer. These experiments support the conclusion that DRM and PDRM represent two stages of a statistical equilibrium between magnetic and perturbing torques: DRM is the first stage that applies at the sediment–water interface, and PDRM is the later stage that develops inside the more strongly perturbed mixed layer. Sedimentation rate and the kinetics of particle reorientation through rotational diffusion determines whether DRM survives the new equilibrium or whether it is replaced by a PDRM, producing natural remanent magnetizations (NRMs) with intermediate intensity. According to the (P)DRM efficiencies measured in our redeposition experiments (Fig. 5b), such intensity variations could amount to a factor of ∼2. Larger effects might be expected in naturally deposited sediment, where higher shear strengths, and thus larger values of *τ*_p_, further reduce the PDRM intensity. For example, a positive correlation was found between sedimentation rate and NRM intensity in pelagic sediments of the eastern equatorial Pacific Ocean^30^. The effect of salinity on flocculation^31^ is another factor that must be considered when extrapolating our (P)DRM intensities to natural sediments.

This new quantitative understanding of how sediment becomes magnetized in the Earth's magnetic field will hopefully facilitate development of better techniques for paleointensity reconstructions, especially if proxies for bioturbation activity and the acquisition efficiency of specific magnetic mineral components can be used. For example, the recently gained awareness of the widespread preservation of magnetofossils in the geological record^32^ raises questions about their contribution to NRM, in comparison with lithogenic magnetic minerals^30^,^33^. Our redeposition experiments have been performed with a magnetofossil-rich sediment and demonstrate that magnetofossils can acquire a PDRM, and, through sediment resuspension (Fig. 1a), even a DRM. In this regard, the NRM acquisition process of magnetofossils might not differ substantially from that of terrigenous inputs. On the other hand, magnetofossils can have a different NRM acquisition efficiency^33^ compared with terrigenous magnetic minerals, depending on the size of sediment particles to which they might adhere once the biological material surrounding magnetosome chains is dissolved. In this case, NRM acquisition efficiencies are expected to depend on sediment mineralogy in a reproducible manner, so that possible variations can be corrected, along with the effects of sedimentation and bioturbation rates.

## Methods

### Sediment collection and characterization

Freshly collected pond sediment was transferred to glass aquaria at room temperature^34^. A stable chemical stratification formed spontaneously within ∼6 days, as seen from microelectrode oxygen measurements and magnetotactic bacteria counts^19^,^20^. Sediment slurries for redeposition experiments were taken from the uppermost ∼3 cm of aquaria after different storage periods and were subjected to further treatments (groups D and E) or were immediately prepared for redeposition experiments (groups A–C). Dissolved oxygen profiles were measured with a microsensor mounted on a micromanipulator (Unisense). Sediment grain size distributions were measured with a Beckman Coulter LS230 laser diffractometer (Earth Sciences Department, Ludwig-Maximilians University, Germany) on aqueous suspensions that were not subjected to dispersion treatments, owing to the fact that only the size of grains and grain aggregates behaving as solid units is relevant for sedimentary remanence acquisition.

The magnetic mineralogy of collected material was investigated with high-resolution first-order reversal curves^35^ acquired with a PMC MicroMag 3900 vibrating sample magnetometer (Institute of Rock Magnetism, University of Minnesota, USA), using a standard protocol for sediment characterization^36^,^37^. Relative magnetic contributions from primary and secondary minerals have been estimated on the basis of the difference between identical first-order reversal curves measurements before and after selective chemical dissolution of ultrafine (<300 nm) iron oxides with a citrate–bicarbonate–dithionite solution^38^ (Supplementary Fig. 3, Supplementary Table 1).

### Redeposition experiments

Redeposition experiments were performed in homogeneous magnetic fields controlled by two sets of Helmholtz coils. In all, 5 ml sediment slurry aliquots were mixed with 10 ml water and were sealed in glass vials with 22-mm diameter. The vials have a negligible (∼0.26 nAm^2^) magnetic moment compared with that acquired by the sediment (>20 nAm^2^ in 20 μT). Sets of vials for DRM acquisition were immediately placed in the Helmholtz coils after vigorous stirring and exposed to field intensities of 0–150 μT with 0–80° inclination, depending on the experimental setting. Control experiments for checking the acquisition/decay of viscous magnetizations were performed under identical conditions using dried sediment powder pressed into plastic containers typically used for palaeomagnetic experiments. In this case, magnetic particles are mechanically blocked during the whole experiment, and any magnetization change is imputable only to magnetic viscosity. The procedure used for PDRM acquisition was identical, except for initial storage of the filled vials in a magnetically shielded laboratory for ∼5 days, so that deposition occurred in a nearly null field (<0.5 μT).

Magnetic measurements were performed with a three-axis, vertical bore 2G Enterprises superconducting rock magnetometer (Earth Sciences Department, Ludwig-Maximilians University) located in a shielded laboratory with <0.5 μT average residual field^26^. For this purpose, vials were removed from the Helmholtz coils and were lowered into the measurement position of the magnetometer, carefully avoiding tilting and mechanical shock. After each measurement, which lasted for ∼10 min, the vials were returned to their previous orientation inside the Helmholtz coils to continue the experiment.

### Microbiological investigations

The group E vials were prepared by adding a broad-spectrum bactericide mix consisting of 20 μl aliquots of chloromycetin (30 g l^−1^), ampicillin (100 g l^−1^), streptomycin (50 g l^−1^) and kanamycin (50 g l^−1^). Although microbial activity is not completely eliminated by this treatment^39^, it has the distinct advantage that it does not alter the sediment as chemical treatments would. Viable bacteria were enumerated after each redeposition experiment using the spread plate method^40^. For this purpose, 100 μl was taken from each vial and diluted with 3.9 ml of sterilized water. After homogenization, 10 μl of the diluted suspension was mixed with 80 μl of sterilized water and was evenly spread on a Lysogeny broth agar plate with a culture medium consisting of 1% w/v tryptone, 0.5% w/v yeast extract, 1% w/v NaCl and water. Agar plates (3 for each vial) were incubated at 37 °C for 16 h and visible colony-forming units were counted.

## Additional information

**How to cite this article:** Zhao, X. *et al*. Microbially assisted recording of the Earth's magnetic field in sediment. *Nat. Commun.* 7:10673 doi: 10.1038/ncomms10673 (2016).

## Supplementary Material

Supplementary Information Supplementary Figures 1-3, Supplementary Table 1, Supplementary Note 1 and Supplementary References
