# Supplementary material for: Microbially assisted recording of the Earth's magnetic field in sediment
Source: Nat Commun. 2016 Feb 11;7:10673. doi: 10.1038/ncomms10673 (PMC4753249; doi:10.1038/ncomms10673)
Supplement: Supplementary Information — Supplementary Figures 1-3, Supplementary Table 1, Supplementary Note 1 and Supplementary References [file ncomms10673-s1.pdf]

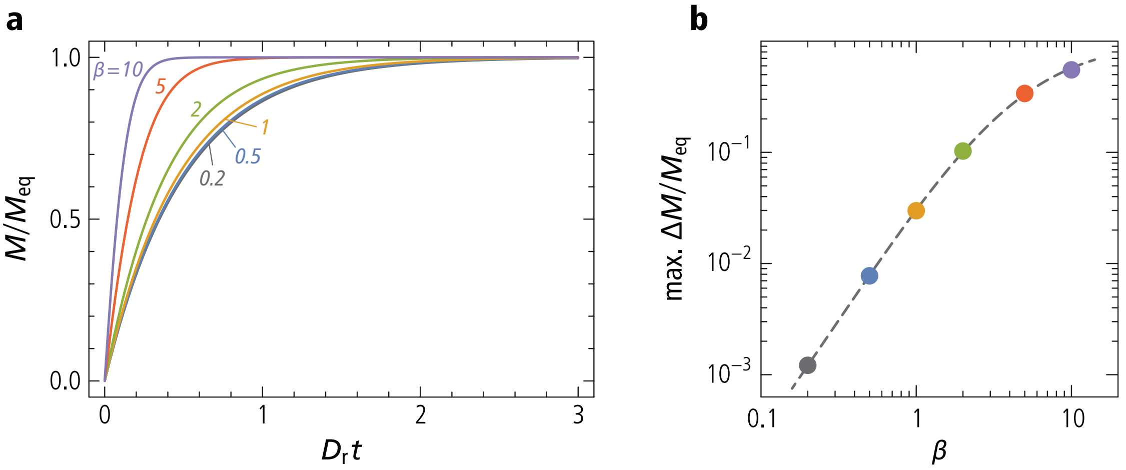

Supplementary Figure 1 | Field dependence of PDRM acquisition curves. (**a**) Calculated PDRM acquisition curves for $\beta = 0.2$, 0.5, 1, 2, 5, and 10, where $\beta = mB/(D\Gamma)$. All curves are normalized by the equilibrium magnetization $M_{eq} = M(t = \infty)$. (**b**) Maximum difference ΔM between PDRM acquisition curves shown in (a) for selected values of β and the limit case given by $\beta \to 0$.

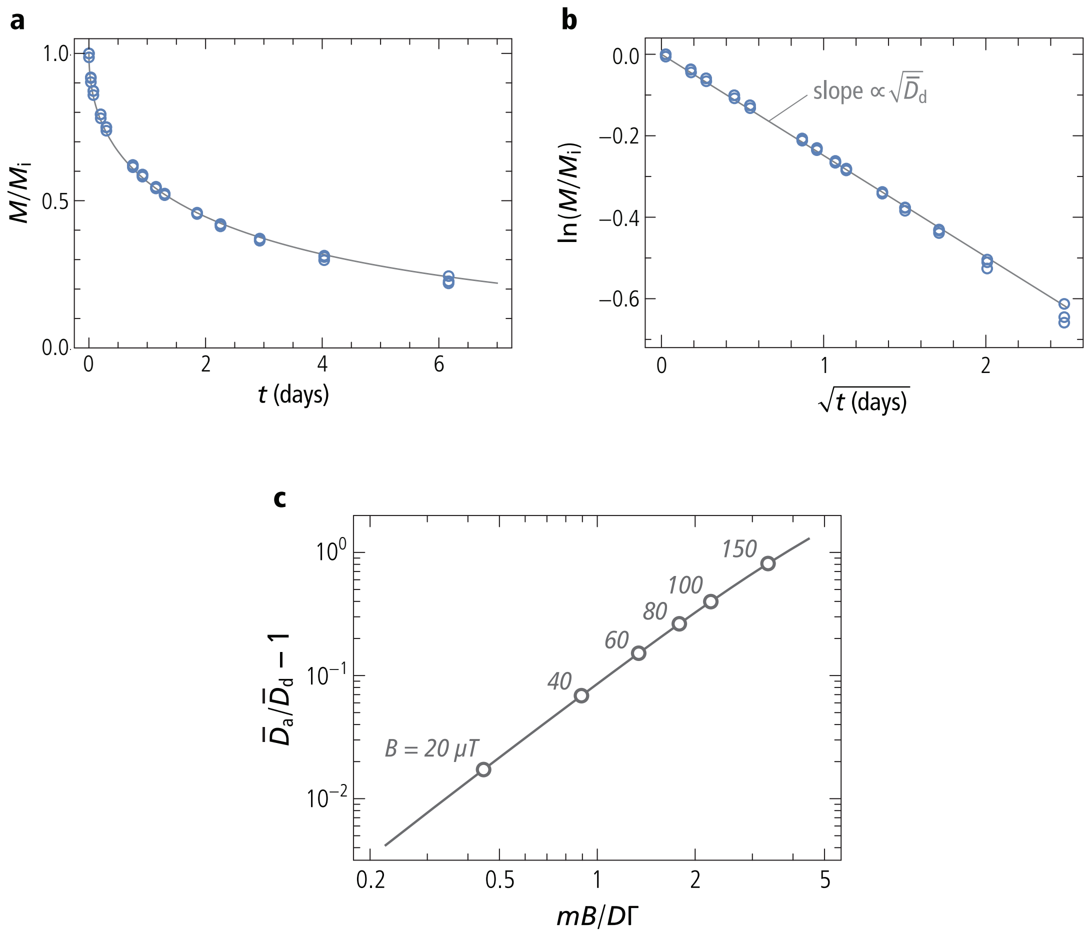

Supplementary Figure 2 | Reconstructing the distribution of rotational diffusion coefficients from PDRM decay curves. (**a**) PDRM decay curves for three samples of group A (blue circles), normalized by the initial value M_i, and least squares fit with $\tilde{f}_\mathrm{d} = \exp(-b\sqrt{t})$ (grey line). (**b**) Same as (a) for the logarithm of M/M_i vs. the square root of decay time. The slope b is proportional to the square root of the median rotational diffusion coefficient $\bar{D}$. (**c**) Ratio between the median rotational diffusion coefficients deduced from acquisition curves (D_a) and decay curves (D_d) calculated from numerical solutions of the Debye-Smoluchowski equations, as a function of the Boltzmann factor $mB/(D\Gamma)$. Boltzmann factors corresponding to fields used in PDRM experiments (dots) have been deduced from the Langevin fit of PDRM vs. applied field data shown in Figure 5b, i.e. $M_\mathrm{eq} \propto S(B/B_0)$ with B_0 = 26.7 μT.

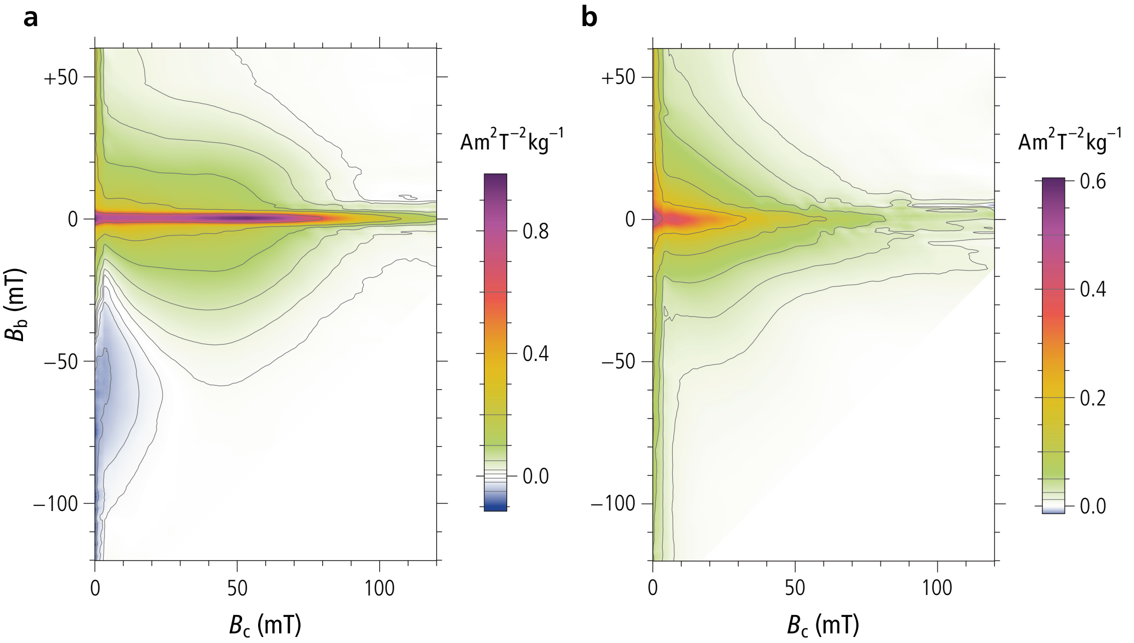

Supplementary Figure 3 | FORC diagrams of sediment used in redeposition experiments. FORC diagrams for untreated (**a**) and chemically treated (**b**) pond sediment, where secondary iron oxides, including magnetofossils, have been selectively dissolved. The central ridge along $B_b = 0$ and negative contributions in the lower left part of the diagram in (a) are unique signatures of single-domain particles and magnetofossils.

Supplementary Table 1 | Magnetizations derived from FORC measurements. M_{FORC} is the total magnetization obtained by integrating the FORC distribution, M_{rs} is the saturation remanence, and M_{cr} the magnetization obtained by integration of the central ridge. All magnetizations are given in $\mathrm{mAm^2kg^{-1}}$.

	M_{FORC}	M_{rs}	M_{cr}
Untreated sediment	1.62 ± 0.01	1.37 ± 0.002	0.40 ± 0.005
Treated sediment	0.90 ± 0.005	0.53 ± 0.001	0
Difference	0.72	0.84	0.40

Supplementary Note 1

PDRM acquisition kinetics

The magnetization acquired during PDRM experiments is controlled by the statistical distribution $p(t,\theta,\varphi)$ of magnetic grain orientations at time t, where orientations are expressed in spherical coordinates by the angle θ between magnetic moment vector and applied field direction, and the azimuthal angle φ. This distribution obeys the Debye-Smoluchowski equation:

$$\frac{\partial p}{\partial t} = D\Delta p + \frac{1}{\Gamma}\nabla(p\nabla V), \tag{1}$$

where D is the rotational diffusion coefficient, Γ is the rotational viscous drag coefficient (e.g. $\Gamma = 8\pi\eta a^3$ for spheres with radius a immersed in a fluid with dynamic viscosity η), and V is a potential whose gradient defines a torque $\tau = -\nabla V$ that adds to the random torques associated with diffusion[1]. The potential V is the sum of (1) a systematic term $-m_i B\cos\theta$, which yields the magnetic torque experienced by a particle with magnetic moment m_i in the applied field B, and (2) a random "holding potential" U_i that accounts for mechanical interaction forces between particles. The general solution of equation (1) at equilibrium (i.e., $\partial p/\partial t = 0$) is the Boltzmann distribution:

$$p_{\mathrm{eq}}(\theta,\varphi) = p_0 \exp\left[\frac{-V(\theta,\varphi)}{D\Gamma}\right], \tag{2}$$

where p_0 is a constant ensuring that the total probability associated with p_{eq} is 1.

The overall effect of the holding potential is equivalent to an increase of the viscous drag[1]. Therefore, $V = -mB\cos\theta$ can be identified with the effective total potential if Γ is corrected for the effect of holding torques, and equation (1) can be rewritten as

$$\frac{1}{D}\frac{\partial p}{\partial t} = \frac{1}{\sin\theta}\frac{\partial}{\partial\theta}\left[\frac{\partial p}{\partial\theta}\sin\theta + \beta p\sin^2\theta\right], \tag{3}$$

where $\beta = mB/(D\Gamma)$ is the so-called Boltzmann factor. Upon substituting $x = \cos\theta$ and $t' = Dt$ we obtain the dimensionless form

$$\frac{\partial p}{\partial t'} = \frac{\partial}{\partial x}\left[(1-x^2)\left(\frac{\partial p}{\partial x} - \beta p\right)\right] \tag{4}$$

of the Debye-Smoluchowski equation, which we solve numerically in order to reproduce PDRM acquisition (i.e., $\beta > 0$) and decay (i.e., $\beta = 0$) experiments. The associated magnetization is obtained by integrating the magnetic moment components along the direction of B, i.e.:

$$\frac{M(t)}{M_0} = 2\pi\int_{\theta=0}^{\pi} p(t,\theta)\cos\theta\sin\theta\,\mathrm{d}\theta, \tag{5}$$

where M_0 is the magnetization corresponding to fully aligned magnetic moments.

A general analytical solution of equation (4) exists only for $\beta = 0$. In this case

$$p(t,\theta) = \frac{1}{4\pi} + \frac{1}{4\pi}\sum_{l=1}^{\infty} c_l e^{-Dl(l+1)t} P_l(\cos\theta), \tag{6}$$

where P_l are Legendre polynomials of order l, and c_l are coefficients determined by the initial distribution $p(0,\theta)$. Insertion of this solution into equation (5) gives[2]:

$$\frac{M(t)}{M_0} = \frac{1}{2} c_1 e^{-2Dt} \int_{\theta=0}^{\pi} P_1(\cos\theta)\cos\theta\sin\theta\,\mathrm{d}\theta = \frac{c_1}{3} e^{-2Dt}. \tag{7}$$

Accordingly, any initial magnetization decays exponentially in zero field, regardless of how it was acquired (i.e. regardless of the initial distribution of magnetic moment orientations).

On the other hand, PDRM acquisition curves can be obtained only by numerical solution of equation (4). Acquisition curves originating from a fully randomized initial state (i.e. $p(0,\theta) = 1/4\pi$) have been calculated with Wolfram Mathematica® using the following command:

```
NDSolve[{D[p[t,x],t] == D[(1 - x^2)*(D[p[t,x],x] - beta*p[t,x]),x],
         p[0,x] == 1/(4*Pi)},
         p,{t,0,ta},{x,-1,1}]
```

and given values of `beta` for β and `ta` for the maximum acquisition time $t'_\mathrm{a} = Dt_\mathrm{a}$. In the limit case of $\beta \to 0$, numerical solutions converge to

$$\frac{M(t)}{M_0} = \mathfrak{L}(\beta)\left[1 - e^{-2Dt}\right], \tag{8}$$

where $\mathfrak{L}(\beta) = \coth\beta - 1/\beta$ is the Langevin function. Equation (8) is affected by a maximum error of 0.03 as long as $\beta \le 1$. Above this limit, magnetizations are acquired faster than their decay in zero field, due to the increasingly strong aligning torques associated with $\beta > 1$ (Supplementary Figure 1).

In the case of PDRM acquired in weak fields (i.e. $\beta < 1$), the following relationship holds between the acquisition curve M_a and the zero-field decay curve M_d:

$$M_\mathrm{a} = M_\mathrm{eq} - M_\mathrm{eq}\frac{M_\mathrm{d}}{M_\mathrm{f}}, \tag{9}$$

where $M_\mathrm{eq} = M_\mathrm{a}(t \to \infty)$ is the equilibrium magnetization in the applied field, and $M_\mathrm{f} = M_\mathrm{a}(t_\mathrm{a})$ is the "final" PDRM acquired over time t_a. Solution of equation (9) with respect to M_d gives

$$\frac{M_\mathrm{d}}{M_\mathrm{f}} = 1 - \frac{M_\mathrm{a}}{M_\mathrm{eq}} = 1 - s\frac{M_\mathrm{a}}{M_\mathrm{f}}, \tag{10}$$

where $s = M_\mathrm{f}/M_\mathrm{eq}$ is the fraction of M_eq reached at the end of the acquisition time. This parameter is estimated using an appropriate model of acquisition/decay curves based on a distribution of rotational diffusion coefficients.

Modelling PDRM acquisition/decay curves

As shown above, weak-field PDRM acquisition and decay curves inside a mixed sediment layer characterized by a rotational diffusion coefficient D are given by:

$$\frac{M_{\mathrm{a}}(t)}{M_{\mathrm{eq}}}=1-e^{-2Dt}$$
$$\frac{M_{\mathrm{d}}(t)}{M_{\mathrm{d}}(t=0)}=e^{-2Dt}, \tag{11}$$

where M_{eq} is the magnetization at equilibrium with the applied field. Because D is controlled by several factors, including particle size, bioturbated sediment is modelled by a distribution of D values defined by a probability density function $p_{\mathrm{r}}(D)$. Accordingly, each magnetized grain is subjected to its own rotational diffusion process characterized by an exponential acquisition and decay curve of the form given by equation (11). Integration of $(1-e^{-2Dt})p_{\mathrm{r}}$ over D gives the normalized acquisition curve

$$\frac{M_{\mathrm{a}}(t)}{M_{\mathrm{eq}}}=\int_0^\infty p_{\mathrm{r}}(D)(1-e^{-2Dt})\mathrm{d}D=1-\int_0^\infty p_{\mathrm{r}}(D)e^{-2Dt}\mathrm{d}D. \tag{12}$$

In real PDRM acquisition experiments, acquisition is stopped at time t_{a} before full equilibrium with the applied field is reached (i.e. $M_{\mathrm{a}}(t_{\mathrm{a}})/M_{\mathrm{eq}}=s<1$), so that the PDRM contribution of grains with rotational diffusion coefficient D is proportional to $p_{\mathrm{r}}(D)(1-e^{-2Dt_{\mathrm{a}}})$. The decay of the total PDRM in zero field is thus given by

$$\frac{M_{\mathrm{d}}(t)}{M_{\mathrm{eq}}}=\int_0^\infty p_{\mathrm{r}}(D)(1-e^{-2Dt_{\mathrm{a}}})e^{-2Dt}\mathrm{d}D. \tag{13}$$

Equations (12-13) can be rewritten as

$$\frac{M_{\mathrm{a}}(t)}{M_{\mathrm{eq}}}=1-f_{\mathrm{d}}(t)$$
$$\frac{M_{\mathrm{d}}(t)}{M_{\mathrm{eq}}}=f_{\mathrm{d}}(t)-f_{\mathrm{d}}(t_{\mathrm{a}}+t), \tag{14}$$

where

$$f_{\mathrm{d}}(t)=\int_0^\infty p_{\mathrm{r}}(D)e^{-2Dt}\mathrm{d}D \tag{15}$$

is the normalized decay curve of the equilibrium PDRM (i.e. the magnetization acquired with $t_{\mathrm{a}} \to \infty$). In mathematical terms, $f_{\mathrm{d}}(2t)$ is the Laplace transform of $p_{\mathrm{r}}(D)$. Conversely, the probability distribution p_{r} is determined uniquely by the inverse Laplace transform

$$p_{\mathrm{r}}(D)=\frac{1}{2\pi i}\lim_{T\to\infty}\int_{\omega-iT}^{\omega+iT} f_{\mathrm{d}}(2t)e^{Dt}\mathrm{d}t, \tag{16}$$

where ω is a real parameter chosen to avoid singularities of f_{d} in the complex plane.

In principle, equations (14-16) can be used to reconstruct p_r from a set of PDRM acquisition/decay experiments, provided that $p_r(D)$ does not change significantly over the experiment duration. In order to test the validity of this condition, acquisition and decay curves are fitted independently using a suitable parameterized approximation $\tilde{f}_d$ of f_d for which the inverse Laplace transform is known. The model function $\tilde{f}_d$ must provide a good fit of all experimental data with a minimum number of parameters; ideally a single one representing the median of $p_r(D)$. In order to guess a suitable analytical expression for $\tilde{f}_d$, we plotted the logarithm of normalized decay curves vs. the square root of decay time, obtaining straight lines with different slopes (Supplementary Figure 2). These lines are described by $\tilde{f}_d = \exp(-b\sqrt{t})$, where b is the line slope on the logarithmic plot. The inverse Laplace transform of $\tilde{f}_d$ is the probability density function

$$p_r(D, D_0) = \sqrt{\frac{\xi \bar{D}}{\pi}} D^{-3/2} e^{-\xi \bar{D}/D}, \tag{17}$$

with $\xi = 0.227$, associated cumulative distribution

$$P_r(D, \bar{D}) = 1 - \mathrm{erf}\left[\sqrt{\frac{\xi \bar{D}}{D}}\right], \tag{18}$$

and median $\bar{D}$. This probability function yields the decay curve

$$\tilde{f}_d(t, \bar{D}) = e^{-2\sqrt{2\xi \bar{D} t}}, \tag{19}$$

which is used to fit all experimental data.

Since M_{eq} is unknown, acquisition and demagnetization curves are normalized by the maximum PDRM, i.e. $M_f = M_a(t_a)$, instead of M_{eq}, and models include an (unknown) “saturation” factor $s = M_f/M_{eq}$, so that

$$\begin{aligned} f_a &= \frac{M_a(t)}{M_f} = \frac{1}{s}\left[1 - \tilde{f}_d(t, D_a)\right] \\ f_d &= \frac{M_d(t)}{M_f} = \frac{1}{s}\tilde{f}_d(t, D_d) - \frac{1}{s}\tilde{f}_d\left(\frac{D_a}{D_d}t_a + t, D_d\right) \end{aligned}, \tag{20}$$

where D_a and D_d represent the median rotational diffusion coefficient $\bar{D}$ during PDRM acquisition and decay, respectively. In the case of stationary conditions, $D_a = D_d = D_0$. Each experiment is thus described by three unknown parameters: s, D_a, and D_d. Because of the high sensitivity of these parameters to measurement errors, additional constraints are introduced using the fact that all experiments share the same total acquisition time t_a. In this case, the saturation factor s depends only on D_a and, at least in principle, on the intensity of the field applied during acquisition. As discussed above, strong applied fields speed the acquisition process and are equivalent to an apparent increase of D_a. Therefore, D_a-values obtained from fitting acquisition curves with equation (20) need to be corrected *a-posteriori*, as described later. Finally, s is sensitive to large variations in the rotational diffusion coefficient, such as those existing between sample groups A-

E, while samples belonging to the same groups are characterized by minor differences of D_a and are conveniently modelled with a common value of s, i.e., s_A for group A, s_B for group B, and so on. Small corrections of s can be applied *a-posteriori* once the modelled acquisition curves are calculated. The model parameters $D_{a,i}$, $D_{d,i}$, and s_l for the *i*-th sample belonging to group *l* are determined by minimization of the sum of squared model residuals

$$\varepsilon = \sum_{i,k}\left[f_{a,k}(t_{ik})-1+\frac{1}{s_l}\tilde{f}_d(t_{ik},D_{a,i})\right]^2 + \sum_{i,k}\left[f_{d,k}(t_{ik})-\frac{1}{s_l}\tilde{f}_d(t_{ik},D_{d,i})+\frac{1}{s_l}\tilde{f}_d\left(\frac{D_{a,i}}{D_{d,i}}t_a+t_{ik},D_{d,i}\right)\right]^2, \tag{21}$$

where t_{ik} is the time corresponding to the *k*-th measurement of the *i*-th decay or acquisition curve, respectively, and $l =$ A, B, C, D, and E. The model is subsequently refined by calculating the saturation factors

$$s_i = 1-\tilde{f}_d(t_a,D_{a,i}) \tag{22}$$

and minimizing

$$\varepsilon = \sum_{i,k}\left[f_{a,k}(t_{ik})-1+\frac{1}{s_i}\tilde{f}_d(t_{ik},D_{a,i})\right]^2 + \sum_{i,k}\left[f_{d,k}(t_{ik})-\frac{1}{s_i}\tilde{f}_d(t_{ik},D_{d,i})+\frac{1}{s_i}\tilde{f}_d\left(\frac{D_{a,i}}{D_{d,i}}t_a+t_{ik},D_{d,i}\right)\right]^2 \tag{23}$$

with respect to $D_{a,i}$ and $D_{d,i}$. Stationary conditions are expected to yield $D_{a,i} = D_{d,i}$ after correcting $D_{a,i}$ for the effect of the acquisition field intensity. The correction factors have been determined from numerical solutions of the Debye-Smoluchowski equation, and are shown in Supplementary Figure 2c for the 20, 40, 60, 80, 100, and 150 μT fields used in our experiments.

Magnetic characterization of sediment

Sediment from the Niederlippach pond is known to contain abundant magnetotactic bacteria, as documented by microscopy observations[3–5]. A combination of selective chemical extraction and magnetic measurements were used to quantify the relative contribution of magnetofossils to the remanent magnetization of sediment.

The chemical extraction procedure used for the selective dissolution of secondary iron oxides, including magnetofossils, is described in ref. 6. The magnetic properties of pond sediment before and after selective chemical extraction were investigated with high-resolution (0.5 mT field steps) FORC measurements[7] for a total of 450 curves ranging from −0.16 to +0.19 T. A total of six measurement sets for each sample were averaged and processed with VARIFORC[8] and results are shown in Supplementary Figure 3. The FORC diagram of the untreated sediment contains a so-called central ridge[9], which is a unique signature of isolated single-domain particles or linear chains

of such particles produced by magnetotactic bacteria[9–11]. This signature disappears upon chemical extraction, which means that the single-domain particles contributing to the central ridge are not protected from dissolution by inclusion in other host minerals, therefore excluding a lithogenic origin. The FORC diagram of the chemically treated sample, on the other hand, is typical of detrital sources containing pseudo-single-domain particles[12–13].

The relative contribution of chemically extracted magnetic particles can be quantified by considering the difference between pre- and post-extraction magnetizations. Of particular interest is the total FORC magnetization, M_{FORC}, which represents the contribution of all irreversible magnetic processes, and the saturation remanence M_{rs}, which represents the maximum magnetization of particles in a null field. The latter is particularly relevant for (P)DRM acquisition experiments. In this case, the relative contribution of extractable magnetic minerals amounts to 61% (Supplementary Table 1).

The magnetic contribution of live magnetotactic bacteria to M_{rs} can be estimated from cell counts. Maximum cell concentrations of 300 cells/μl for this type of sediment refer to magnetotactic bacteria performing polar magnetotaxis, which have been counted by the hanging drop method[3–4]. Assuming a similar amount of bacteria performing axial magnetotaxis, which would not be detected by the hanging drop method, we assume a maximum cell concentration of 600 cells/μl. The total magnetic moment of individual cells can be calculated from the average number of magnetosomes, and is comprised between ~1 and ~10 fAm^2 (ref. 4). Assuming a mean magnetic moment of 5 fAm^2/cell, we obtain a maximum volume-normalized magnetization of 1.5 mA/m, which, for a sediment with 88% porosity[4], converts to a mass-normalized magnetization of 4.5 μAm^2/kg for the dry sediment. This corresponds to <1% of the saturation remanence attributable to magnetofossils.

Supplementary References

1. Egli, R. & Zhao, X. Natural remanent magnetization acquisition in bioturbated sediment: general theory and implications for relative paleointensity reconstructions. *Geochem. Geophys. Geosyst.* **16**, 995–1016 (2015).

2. Perrin, F. The Brownian movement of an ellipsoid (I) – The dielectric dispersion of ellipsoidal molecules, *J. Phys. Radium*, *5*, 497–511 (1934).

3. Jogler, C. *et al.* Cultivation-independent characterization of '*Candidatus* Magnetobacterium Bavaricum' via ultrastructural, geochemical, ecological and metagenomic methods, *Environ. Microbiol.* **12**, 2466–2478 (2010).

4. Mao, X., Egli, R., Petersen, N., Hanzlik, M. & Zhao, X. Magnetotaxis and acquisition of detrital remanent magnetization by magnetotactic bacteria in natural sediment: First experimental results and theory, *Geochem. Geophys. Geosyst.* **15**, 255–283 (2014).

5. Mao, X., Egli, R., Petersen, N., Hanzlik, M. & Liu, X. Magneto-chemotaxis in sediment: first insights, *PLOS One* **9**, e102810 (2014).

6. Ludwig, P. *et al.* Characterization of primary and secondary magnetite in marine sediment by combining chemical and magnetic unmixing techniques, *Global Planet. Change* **110**, 321–339 (2013).

7. Pike, C. R., Roberts, A. P. &Verosub, K. L. Characterizing interactions in fine magnetic particle systems using first order reversal curves. *J. Appl. Phys.* **85**, 6660–6667 (1999).

8. Egli, R. VARIFORC: An optimized protocol for calculating non-regular first-order reversal curve (FORC) diagrams, *Global Planet. Change* **110**, 302–320 (2013).

9. Egli, R., Chen, A. P., Winklhofer, M., Kodama, K. P. & Horng, C. S. Detection of noninteracting single domain particles using first-order reversal curve diagrams, *Geochem. Geophys. Geosyst.* **11**, Q01Z11 (2010).

10. Newell, A. J. A high-precision model of first-order reversal curve (FORC) functions for single-domain ferromagnets with uniaxial anisotropy. *Geochem. Geophys. Geosyst.* **6**, Q05010 (2005).

11. Heslop, D., Roberts, A. P. & Chang, L. Characterizing magnetofossils from first-order reversal curve (FORC) central ridge signatures, *Geochem. Geophys. Geosyst.* **15**, 2170–2179 (2014).

12. Roberts, A. P., Pike, C. R. & Verosub, K. L. First-order reversal curve diagrams: A new tool for characterizing the magnetic properties of natural samples. *J. Geophys. Res.* **105**, 28461–28475 (2000).

13. Muxworthy, A. R. & Dunlop, D. J. First-order reversal curve (FORC) diagrams for pseudo-single-domain magnetites at high temperature. *Earth Planet. Sci. Lett.* **203**, 369–382 (2002).
